# ErbB3 mRNA leukocyte levels as a biomarker for major depressive disorder

**DOI:** 10.1186/1471-244X-12-145

**Published:** 2012-09-18

**Authors:** Elena Milanesi, Alessandra Minelli, Nadia Cattane, Annamaria Cattaneo, Cristina Mora, Alessandro Barbon, Alessandra Mallei, Maurizio Popoli, Vincenzo Florio, Andreas Conca, Stefano Bignotti, Massimo Gennarelli

**Affiliations:** 1 Department of Biomedical Sciences and Biotechnologies - Biology and Genetic Division, University of Brescia, Viale Europa 11, Brescia, 25123, Italy; 2 Department of Pharmacological Sciences, Center of Neuropharmacology, University of Milano, Via Balzaretti 9, Milano, Italy; 3 Department of Psychiatry, Central Hospital of Bolzano, Bolzano, Italy; 4 Psychiatric Unit, I.R.C.C.S. “San Giovanni di Dio” - Fatebenefratelli, via Pilastoni 4, Brescia, 25123, Italy; 5 Genetic Unit, I.R.C.C.S. “San Giovanni di Dio” - Fatebenefratelli, Via Pilastroni, 4, Brescia, 25123, Italy

**Keywords:** Major Depressive Disorder MDD, ErbB3, Fgfr1, Biomarkers, Leukocytes

## Abstract

**Background:**

In recent years, the identification of peripheral biomarkers that are associated with psychiatric diseases, such as Major Depressive Disorder (MDD), has become relevant because these biomarkers may improve the efficiency of the differential diagnosis process and indicate targets for new antidepressant drugs. Two recent candidate genes, *ErbB3* and *Fgfr1*, are growth factors whose mRNA levels have been found to be altered in the leukocytes of patients that are affected by bipolar disorder in a depressive state. On this basis, the aim of the study was to determine if *ErbB3* and *Fgfr1* mRNA levels could be a biomarkers of MDD.

**Methods:**

We measured by Real Time PCR ErbB3 and Fgfr1 mRNA expression levels in leukocytes of MDD patients compared with controls. Successively, to assess whether ErbB3 mRNA levels were influenced by previous antidepressant treatment we stratified our patients sample in two cohorts, comparing drug-naive versus drug-free patients. Moreover, we evaluated the levels of the transcript in MDD patients after 12 weeks of antidepressant treatment, and in prefrontal cortex of rats stressed and treated with an antidepressant drug of the same class.

**Results:**

These results showed that *ErbB3* but not *Fgfr1* mRNA levels were reduced in leukocytes of MDD patients compared to healthy subjects. Furthermore, *ErbB3* levels were not affected by antidepressant treatment in either human or animal models

**Conclusions:**

Our data suggest that ErbB3 might be considered as a biomarker for MDD and that its deficit may underlie the pathopsysiology of the disease and is not a consequence of treatment. Moreover the study supports the usefulness of leukocytes as a peripheral system for identifying biomarkers in psychiatric diseases.

## Background

Major Depressive Disorder (MDD) is a disabling psychiatric condition that is among the top five leading causes of disability and disease burden throughout the world [1]. MDD is a complex disease that is characterised by the interaction between genetic and environmental factors, and its hereditability, as assessed by twin and adoption studies, is approximately 40-50% [2].

A biomarker is defined as a characteristic that is objectively measured and evaluated as an indicator of a normal biological process, a pathogenic process, or pharmacologic responses to a therapeutic intervention [3]. The search for MDD biological markers could be important for differential diagnosis, the optimisation of patient care and for the development of more effective drug treatments. During the past several years, research has focused on the identification of MDD peripheral biomarkers, supporting the idea that the neuronal alterations of this disabling disorder and its response to treatment might be reflected in peripheral biological systems such as blood [4].

Moreover, clinical studies have demonstrated that patients with MDD and several pharmacological and non-pharmacological treatments are associated with altered blood/serum levels of growth factors such as brain-derived neurotrophic factor (BDNF) [5,6], insulin-like growth factor-1 (IGF-1), vascular endothelial growth factor (VEGF) [7], glial cell line derived neurotrophic factor (GDNF) and fibroblast growth factor-2 (FGF-2) [4].

Using an approach termed convergent functional genomics (CFG), which translationally cross-matches animal models gene expression data with human genetic data and human tissue data (blood, post-mortem brain), Le-Niculescu and colleagues [8] identified a list of candidate blood biomarkers associated with depressive state in patients affected by bipolar disorder. In particular, they found five genes involved in growth factor signalling (Fgfr1, ErbB3, Igfbp4, Igfbp6 and Ptprm).

Because gene expression studies on post-mortem brain tissue from depressed patients reported an alteration of Fgfr1 [9] and ErbB3 mRNA levels [10], and both growth factors can be easily measured in blood, we focussed our attention on these genes.

Fgfr1 is a member of the fibroblast growth factor receptor **(**FGFR**)** family; these transmembrane catalytic receptors have intracellular tyrosine kinase activity and play significant roles in the development and maintenance of the central nervous system (CNS) [11]. Several genome-wide gene expression microarray analyses of post-mortem brains that were obtained from patients affected by MDD or suicide victims reported a dysregulation of the FGF system, suggesting a role for the FGFR family in the pathogenesis of MDD [9].

V-erb-b2 erythroblastic leukaemia viral oncogene homolog 3 (ErbB3) is a tyrosine kinase receptor, similar to ErbB2 and ErbB4, that belongs to the epidermal growth factor receptor (EGFR) family. EGFRs are activated by many ligands, and, on the basis of analyses of EGFR-deficient mice, the cell types that are most affected by the absence of EGFR are epithelial and glial cells [12]. Indeed, ErbB3 binds members of the neuregulin family, such as neuregulin 1, and plays an important role in promoting oligodendroglia differentiation [13]. ErbB3 and ErbB2 form homo- or heterodimers that are activated via trans-phosphorylation and phosphorylated. ErbB3:ErbB2 dimers provide docking sites for several factors involved in the initiation of cell signalling pathways, including survival, growth and transformation. In particular, ErbB3 is implicated in myelination processes, controlling the growth and the development of Schwann cells that wrap around nerve axons to provide electrical insulation [14]. A reduction in ErbB3 mRNA expression levels has been found in the temporal cortex of depressed patients [10].

Regarding the role of these two growth factors in antidepressant treatment, studies on animal models of depression indicate that the FGF system may be involved in the response to antidepressants [15], and there are no studies about ErbB3 in rats.

To our knowledge, few studies have investigated biomarkers associated with MDD on leukocytes of drug-naive patients. Because of the antidepressant treatment is a confounding factor influencing the mRNA levels of several transcripts, we consider that the drug-naive state should be the *“conditio sine qua non”* to identify diagnostic biomarkers.

Thus, the aim of this study was to assess whether (1) ErbB3 and FGFR1 mRNA levels are modified in MDD patient leukocytes compared to controls and (2) if their levels could be affected by antidepressant treatments.

## Methods

### Subjects

A total of 26 DSM-IV MDD patients were voluntarily enrolled in the study by the Psychiatry Unit of IRCCS Centro S. Giovanni di Dio FBF, Brescia and by the Department of Psychiatry of Bolzano, Italy; all of the patients signed informed consent forms that were approved by the local Ethics Committee.

Exclusion criteria were the following: a) mental retardation and cognitive disorders; b) a lifetime history, and a family history in first-degree relatives, of schizophrenic, schizoaffective, or bipolar disorders; c) personality disorders, obsessive compulsive disorder, post-traumatic stress disorder, as primary diagnosis; d) comorbidity with eating disorders, substance abuse or dependency.

The biological sampling and clinical evaluations were performed the morning before the antidepressant treatment began and again after 12 weeks of treatment (T12). Blood samples for the expression levels at T12 were available only from 17 patients. Illness severity was assessed by the Montgomery-Åsberg Depression Rating Scale (MADRS).

The sample of 26 patients was clustered into two cohorts; the first, called the “drug-free group”, comprised 13 patients who had been treated previously with one of two antidepressants, an SSRI or TCA. For this reason, before entering in the study, each of the patients had a wash-out period from antidepressant drugs (only low doses of benzodiazepines were allowed) lasting at least 2 weeks. The second, called the “drug-naive group”, was made up of 13 patients who had never had previous treatment with any antidepressant drugs.

The control sample consisted of 19 unrelated healthy volunteers that were screened for DSM-IV Axis I disorder diagnoses using the Mini-International Neuropsychiatric Interview (M.I.N.I.) [16] by expert psychologists. Only healthy volunteers without a history of drug or alcohol abuse or dependence and without a personal or first-degree family history of psychiatric disorders were enrolled in the study. Moreover, an anamnestic schedule was compiled to assess the presence of any medical conditions or pharmacological treatment. All patients and controls were of Italian Caucasian origin and resided in northern Italy. Features of controls and MDD patients are showed in Table 1. 

**Table 1:** Clinical and demographical features of controls and MDD patients

	**Controls (19)**	**MDD patients (26)**	
ERBB3 mRNA levels	1.19 ± 0.74	0.67 ± 0.35	F = 9.44; **p <0.01**
Age (mean ± SD)	54.63 ± 19.58	42.96 ± 8.77	F = 7.29; **p = 0.01**
Gender (N;%female)	17;89%	20;77%	χ2 =1.18, p = 0.28
Education (mean ± SD)	12.7 ± 5.8	11.6 ± 2.7	F = 0.60; p = 0.43

### ErbB3 and Fgfr1 leukocyte gene expression analysis

Two micrograms of total RNA were used for cDNA synthesis using random hexamer primers (Invitrogen) and Superscript II Reverse Transcriptase (Invitrogen) after assessing RNA quality and quantity using a NanoDrop (NanoDrop Technologies, Wilmington, DE). Real Time quantitative RT-PCR analyses were performed using the Step One Real Time System (Applied Biosystems) to determine ErbB3 and Fgfr1 mRNA levels in leukocytes. Taqman probes for the ErbB3 (Hs00176538_m1) and Fgfr1 (Hs00915142_m1) genes were purchased from Applied Biosystems. Target genes mRNA levels have been normalized on the arithmetic mean of β2 microglobulin (B2M; Hs99999907_m1), cytochrome c1 (Cyc1; Hs00357717_m1) and ATP synthase, H+ transporting mitochondrial F1 complex β subunit (Atpb5; Hs00969569). ErbB3 mRNA levels normalized on each housekeeping gene are shown in Additional file 1: Table S1. Each sample was assayed in duplicate and using two independent retrotranscription products. Data analyses were performed according to the comparative Ct method using the Applied Biosystems Real Time software, which automatically determines the optimal baseline and threshold settings via the auto Ct determination feature.

### Footshock stress procedure and drug treatments in animal models

All experimental procedures involving animals were performed in accordance with the European Community Council Directive 86/609/EEC and were approved by Italian legislation on animal experimentation (Decreto Ministeriale 124/2003-A). Sprague–Dawley rats (170–200 g) were used. The footshock (FS)-stress protocol was performed essentially as previously reported [17] (40-min FS-stress; 0.8 mA, 20 min total of actual shock with random intershock length for 2–8 sec). The FS-stress box was connected to a scrambler controller (LE 100–26, Panlab) that delivered intermittent shocks to the metallic floor. Sham-stressed rats (controls) were kept in the stress apparatus without the delivery of shocks.

Two additional groups were considered in the analysis: one group was treated chronically (14 days) with the antidepressant escitalopram (10 mg/kg) and killed 24 h after the last injection; in the second group, rats were subjected to FS-stress 24 h after the last administration and killed 24 h thereafter. For all of the rats, the hippocampus (HPC) and the whole frontal lobe (referred to as prefrontal/frontal cortex (P/FC)) were quickly dissected on ice and processed.

In rat HPC and P/FC, the expression ratio of ErbB3 (RN_00568107) in the treated groups to the control group was calculated using the comparative Ct method as previously described using β-Act *(*Rn00667869_m1), GAPDH (Rn_99999916_s1) and H2AFZ (Rn00821133_g1) transcripts as housekeeping genes. Each individual determination was repeated in duplicate.

### Statistical analysis

Demographic and clinical characteristics in our patient and control samples were described either in quantitative terms of the mean ± standard deviation (SD) or as proportions. Analysis of variance (ANOVA) was used for computing possible differences between groups, whereas the chi-squared (χ2) test was used to evaluate categorical variables. The Pearson coefficient was used to evaluate bivariate correlations. Clinical and biological changes were analysed using a general linear model in a repeated measures design with time (T0, T12) as a within-subjects factor, and the Greenhouse–Geisser correction was applied. When the data were not normally distributed, we used non-parametric tests. Non-parametric comparisons were made with the Wilcoxon signed-rank test.

All analyses were conducted using SPSS Version 17.0 statistical software (SPSS Inc. Chicago, IL).

## Results

### Fgfr1

No correlations were observed between Fgfr1 mRNA levels at baseline and age (p = 0.31), gender (p = 0.85), education (p = 0.16) or illness severity (p = 0.73), and no difference was found in Fgfr1 mRNA levels at T0 between controls and patients (1.14 ± 0.47, 1.09 ± 0.53, respectively; p = 0.76). There were no changes in Fgfr1 mRNA levels after 12 weeks of antidepressant treatment (p = 0.47), and no variations related to symptomatology (p = 0.70) were observed.

### ErbB3

No correlations between T0 Erbb3 mRNA levels and age (p = 0.76; p = 0.52), gender (p = 0.17; p = 0.47), education (p = 0.21; p = 0.32) were found in the whole cohort and in controls sample, respectively. A significant correlation was found with age (p = 0.004) in MDD patients, whereas any effects were observed with gender (p = 0.37), education (p = 0.53) and illness severity (p = 0.08).

**Figure 1:**
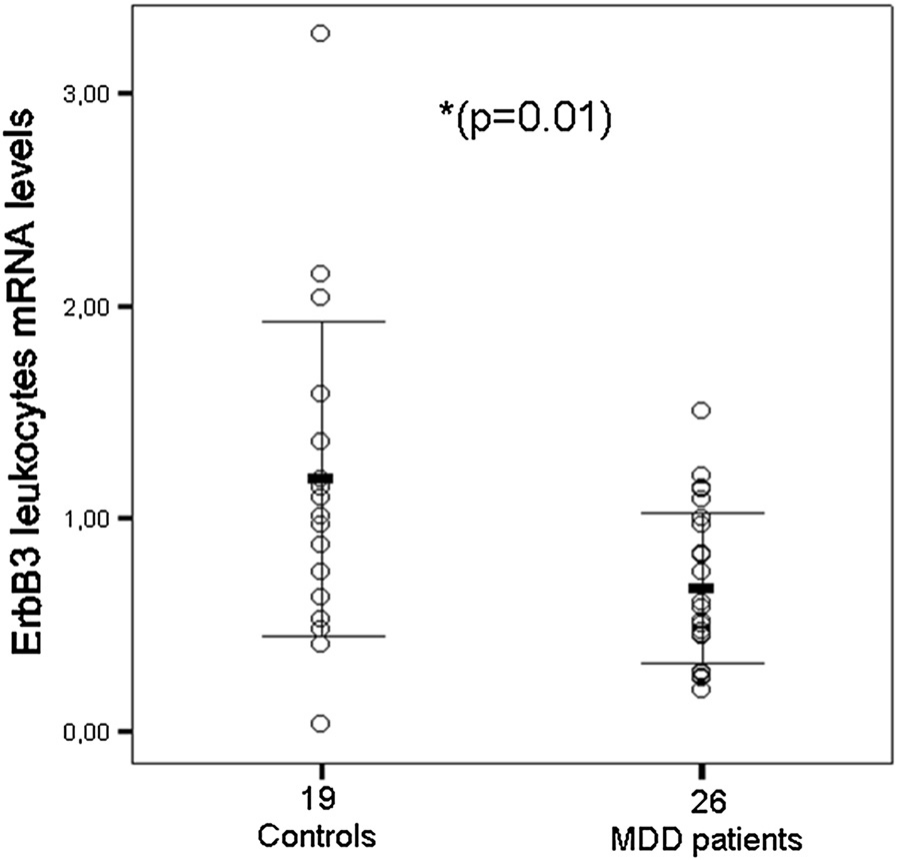
**ErbB3 leukocytes mRNA levels in controls and MDD patients.** Error bars show mean ± 1.0 SD. Expression levels by RT-PCR are the threshold cycle (C_t_) values converted to RNA equivalents and then normalized to geometric mean of housekeeping genes expression.

ErbB3 mRNA levels at baseline were significantly reduced in depressed patients when compared with controls (0.67 ± 0.35, 1.19 ± 0.74, respectively; p = 0.004; Table 1 and Figure 1). The effect remained highly significant after adjusting by age (p = 0.001); the ANOVA showed that the variable age was not significant (p = 0.11) and the Partial Eta Squared Indice estimated the effect size was very low (p = 0.06). On the contrary the Observed Power of the variable group was high (p = 0.93).

Moreover, to evaluate whether an ErbB3 deficit could be influenced by previous antidepressant treatments, we stratified the samples into drug-free and drug-naïve patients. These analyses showed no difference in ErbB3 mRNA levels between the two groups (p = 0.85) (Table 2).

**Table 2:** Clinical and demographical features of the two cohorts of MDD patients

	**Drug-free (13)**	**Drug-naive (13)**	
ERBB3 mRNA levels	0.69 ± 0.44	0.66 ± 0.26	F = 0.04;p = 0.85
Age (mean ± SD)	44.85 ± 9.80	41.08 ± 7.51	F = 1.21; p = 0.28
Gender (N;%female)	12;92%	8;62%	χ2 =3.47, p = 0.06
Education (mean ± SD)	12.0 ± 2.7	11.3 ± 2.8	F = 0.40; p = 0.53
MADRS (mean ± SD)	25.3 ± 6.3	23.3 ± 3.3	F = 1.10; p = 0.30

**Figure 2:**
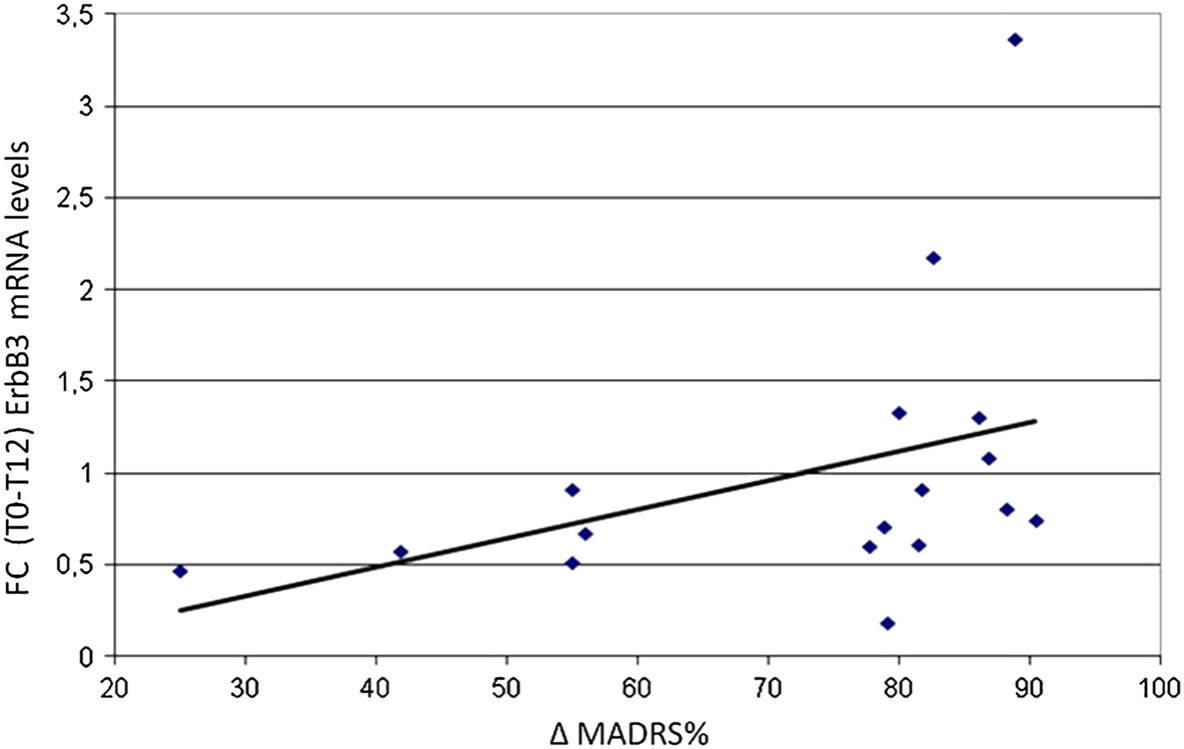
**Correlation between percentage of symptoms improvement ΔMADRS% and Fold changes in ErbB3 mRNA expression (T0-T12).** FC were calculated using the 2^-ΔΔct^ method.

According to the ANOVA, a significant symptomatology decrease occurred at T12 (MADRS scores p <0.001; T0 = 24.06 ± 5.98, T12 = 6.53 ± 4.58), whereas no change in ErbB3 mRNA levels (p = 0.46; T0 = 0.72 ± 0.40, T12 = 0.64 ± 0.51) were found. However, ErbB3 increased at T12 and covaried with the amelioration of depressive symptoms (p = 0.03). A non-parametric Wilcoxon signed rank test indicated a significant correlation between the changes in ErbB3 mRNA levels (as Fold Change using 2^-ΔΔCT^ method) and the percentage of symptoms improvement (p <0.001; mean of ErbB3 changes 1.00 ± 0.76; ΔMADRS 72.68% ± 18.96%; Figure 2). Because two patients were outliers for the changes of ErbB3 we carried out the non-parametric Wilcoxon signed rank test without these two patients and the effect remained significant (p <0.001).

ErbB3 mRNA levels in patients after 12 weeks of antidepressant treatment were still significantly reduced compared to levels of controls (p = 0.02). However, running the analysis including only patients who obtained a relevant amelioration of depressive symptomatology (ΔMADRS >60%) the difference between patients (T12) and controls was lost (p = 0.17).

Finally, no significant alteration in ErbB3 mRNA levels was observed in rat HPC or P/FC, after either the FS-stress protocol or/and the drug treatments (for results see Table 3).

**Table 3:** ErbB3 mRNA levels in rats P/FC and HP

	**P/FC ErbB3 mRNA levels**	**HPC ErbB3 mRNA levels**
Saline vs. Stress	1.01 ± 0.12 vs 0.94 ± 0.10; *p = 0.99*	1.01 ± 0.18 vs 1.04 ± 0.22 *p = 0.99*
Saline vs. Escitalopram	1.01 ± 0.12 vs 0.83 ± 0.18 *p = 0.17*	1.01 ± 0.18 vs 1.02 ± 0.15 *p = 0.99*
Saline vs. Stress + Escitalopram	1.01 ± 0.12 vs 0.90 ± 0.06 *p = 0.99*	1.01 ± 0.18 vs 0.93 ± 0.03 *p = 0.99*

*All comparisons were adjusted for Bonferroni correction.

## Discussion

Our study has identified the involvement of ErbB3 in MDD, showing a reduction in mRNA levels in the leukocytes of depressed patients compared with controls, and that this deficit is not a consequence of antidepressant treatments.

In accord with our study, several other studies have demonstrated that patients with MDD have altered blood/serum levels of growth factors and that chronic stress exposure, which can precipitate or exacerbate depressive episodes, alters the expression of growth factors [4].

By integrating human blood gene expression data, animal model expression data, human genetic linkage/association data and human post-mortem brain data (an approach called convergent functional genomics), Le-Niculescu and colleagues [8] identified a list of blood biomarkers of mood states in bipolar disorders. In particular, they classified the five top-scoring candidate biomarkers for high mood (Mbp, Edg2, Fzd3, Atxn1 and Ednrb) and the five top-scoring candidate biomarkers for low mood (Fgfr1, Mag, Pmp22, Ugt8 and ErbB3). Because Fgfr1 and ErbB3 levels were also found to be altered in post-mortem brains of MDD patients, and their levels can be measured in blood, we investigated the association of mRNA leukocyte levels of these two growth factor genes with the low mood state in a cohort of MDD patients.

Although we did not identify any significant changes in Fgfr1 mRNA levels in leukocytes, an important reduction of ErbB3 mRNA levels emerged in depressed patients, suggesting that ErbB3 could be considered as a biomarker of depression. Moreover, this alteration was not influenced by antidepressant treatments, either in humans or in animal models.

Our findings were in agreement with Aston’s microarray data [10], which found an ErbB3 deficit in the temporal cortex of depressed patients, supporting a role for ErbB3 in the pathogenesis of depression.

Clinically, the use of peripheral biomarkers may be useful for diagnoses and in predicting response to drug treatment. To date, several peripheral biomarkers have been identified; however, some questions must be addressed before the use of biomarkers can be introduced into clinical practice. For MDD biomarkers, it is necessary to clarify if they correspond to a state of depression or a trait (constitutive).

According to the data provided by Le-Niculescu [8] that found low ErbB3 mRNA levels in leukocytes of bipolar patients in a depressive state and the significant correlation we found between the change in the percentages of ErbB3 and change in MADRS, ErbB3 could be considered a biomarker of depressive status. This hyphotesis is supported also by our data showing that ErbB3 levels in patients who obtained a relevant amelioration of depressive symptomatology are not different compared to controls. We could suppose a further increase of ErbB3 levels when patients will be fully remitted.

This study has some limitations. The groups were relatively small and the used samples were limited to leukocytes ones. Moreover, to clarify the role of ERbB3 in a low mood state, new studies on MDD with larger samples and in cohorts of patients with different psychiatric disorders in a depressive state, such as post-traumatic stress disorder (PTSD) or schizophrenia, are necessary.

## Conclusion

Our results on leukocytes in humans and in animal models support the role of ErbB3 in depression and confirm the usefulness of leukocytes as a peripheral model for studying the biochemistry and molecular biology of the central nervous system.

## Abbreviations

MDD: Major Depressive Disorder; BDNF: Brain-derived neurotrophic factor; IGF-1: Insulin-like growth factor-1; VEGF: Vascular endothelial growth factor; GDNF: Glial cell line derived neurotrophic factor; FGF-2: Fibroblast growth factor-2; CFG: Convergent functional genomics; CNS: Central Nervous System; Fgfr1: Fibroblast growth factor receptor-1; ErbB3: V-erb-b2 erythroblastic leukaemia viral oncogene homolog 3; SSRI: Selective serotonin reuptake inhibitors; MADRS: Montgomery-Asberg Depression Scale; DSM-IV: Diagnostic and Statistical Manual of Mental disorders; MINI: Mini international neuropsychiatric interview; TCA: Triciclic antidepressant; B2M: Beta 2 microglobulin; CYC1: Cytochrome C1; ATPb5: ATP synthase H+ transporting mitochondrial F1 complex beta subunit; FS: Footshock; HPC: Hippocampus; P/FC: Prefrontal/Frontal cortex; GAPDH: Glyceraldehyde 3-phosphate dehydrogenase; H2AFZ: H2A histone family, member Z; SD: Standard deviation; PTSD: Post-traumatic stress disorder.

## Competing interests

The authors declare that they have no competing interests.

## Authors’ contributions

EM conceived of the study, participated in its design and the coordination and acquisition of data, performed the statistical analyses, and co-wrote the manuscript; AM participated in its design and the coordination and acquisition of data, screened patients and controls, performed the statistical analyses, and co-wrote the manuscript; NC and AC participated in the design of the study and carried out gene expression analyses; CM and AB carried out gene expression analyses on rats, AM and MP provided the animal models for gene expression analyses; VF, AC, SB enrolled and screened patients; MG conceived of the study, participated in its design and coordination, and helped draft the manuscript and critically reviewed it for intellectual content. All the authors read and approved the final manuscript.

## Pre-publication history

The pre-publication history for this paper can be accessed here:

http://www.biomedcentral.com/1471-244X/12/145/prepub

## Supplementary Material

Additional file 1 Table S1. ErbB3mRNA levels normalized on each housekeeping gene. Click here for file
