# Supplementary material for: ErbB3 mRNA leukocyte levels as a biomarker for major depressive disorder
Source: BMC Psychiatry. 2012 Sep 18;12:145. doi: 10.1186/1471-244X-12-145 (PMC3532413; doi:10.1186/1471-244X-12-145)
Supplement: Additional file 1 — Table S1. ErbB3mRNA levels normalized on each housekeeping gene. [file 1471-244X-12-145-S1.rtf]

Supplementary table
ErbB3mRNA levels normalized on each housekeeping gene
	Controls (19)	MDD patients (26)		
ErbB3 mRNA levels normalized on CYC1	1.35±1.12	0.65±0.40	F=6.32; p=0.004	
ErbB3 mRNA levels normalized on B2M	1.15±0.66	0.70±0.36	F=4.59; p=0.016	
ErbB3 mRNA levels normalized on ATPB5	1.26±0.75	0.74±0.22	F=3.67; p=0.036	
